# Serum hsa-miR-21 expression and its clinical value in pediatric patients with fulminant myocarditis

**DOI:** 10.1016/j.jped.2025.04.007

**Published:** 2025-05-28

**Authors:** Wenwen Wang, Jingmin Sun, Jing Li

**Affiliations:** The First Affiliated Hospital of Anhui Medical University, Department of Pediatrics, Hefei, Anhui Province, China

**Keywords:** Pediatric fulminant myocarditis, Hsa-mir-21, B-type natriuretic peptide, Cardiac troponin I, Creatine kinase isoenzyme

## Abstract

**Objective:**

Fulminant myocarditis (FM) is a distinct and rare form of myocarditis. This study probed hsa-miR-21 expression in FM pediatric patients and its clinical value.

**Methods:**

This study enrolled 88 FM pediatric patients and 90 healthy children (normal controls), with serum sample hsa-miR-21 levels measured by RT-qPCR. FM children were categorized into the good and poor prognosis groups. Correlations of hsa-miR-21 expression with myocardial injury markers [cardiac troponin I (cTnI), creatine kinase isoenzyme (CK-MB)], and independent risk factors and predictive value of hsa-miR-21 expression for FM patients’ poor prognoses were analyzed by Pearson’s, logistic regression, and receiver operating characteristic (ROC) curve analyses.

**Results:**

Serum hsa-miR-21 levels were elevated in FM children relative to the healthy controls and linked with poor prognoses. hsa-miR-21 levels positively correlated with myoglobin (MYO), B-type natriuretic peptide (BNP), cTnI, and CK-MB levels. Elevated hsa-miR-21, CK-MB, left ventricular ejection fraction, C-reactive protein, lactate dehydrogenase, and lactate were independent risk factors for FM children’s poor prognoses. Serum hsa-miR-21 levels yielded an area under the ROC curve of 0.790 in predicting FM pediatric patients’ poor prognoses (58.1 % sensitivity, 87.7 % specificity), with positive and negative predictive values of 74.07 % and 81.97 %, respectively, demonstrating that hsa-miR-21 aided in predicting FM pediatric patients’ poor prognoses to some extent.

**Conclusion:**

Serum hsa-miR-21 was up-regulated in FM pediatric patients, and positively correlated with MYO, BNP, cTnI, and CK-MB. hsa-miR-21 expression was an independent risk factor for FM pediatric patients' poor prognosis, and predicted prognoses to some extent; however, the diagnostic accuracy was limited.

## Introduction

Fulminant myocarditis (FM) is an acute and critical condition of acute myocarditis, accounting for 10 % to 38 % of acute myocarditis. FM represents an inflammatory process that occurs in the heart muscle and leads to an acute episode of heart failure that necessitates support for the pumping function of the heart or emergency management of severe arrhythmias, accompanied by arrhythmias and hemodynamic risks. The diagnosis is assisted by the utilization of cardiac imaging and biomarkers; however, endocardial biopsy continues to be the gold standard.^1^^,^^2^ As a subtype of acute myocarditis, FM is characterized by an insidious onset and rapid progression and is also marked by a sudden and severe diffuse cardiac inflammation, which can result in death due to ventricular arrhythmias, cardiogenic shock, or multiorgan system failure.^3^ Patients with acute viral infection symptoms within 2 to 4 weeks, accompanied by severe hemodynamic abnormalities (pump failure and circulatory failure: heart failure, cardiogenic shock, hypotension, etc.), requiring positive inotropic medications, vasoactive medications or mechanical circulatory support can be diagnosed as FM.^4^ Pediatric FM (PFM) is a highly dangerous condition that can lead to acute heart failure or even sudden death.^5^ The rapid decline in cardiac function in FM patients makes it difficult to obtain an early and clear diagnosis, often resulting in delays or misdiagnosis before the patient’s death,^6^ highlighting the importance of early diagnosis for FM pediatric patients. Presently, the diagnosis of FM relies on a variety of techniques, including echocardiography, electrocardiography, cardiac magnetic resonance imaging, and endomyocardial biopsy.^6^ In the cases of acute onset and a serious threat to the child’s life, it is imperative to initiate the three integrated emergency programs promptly. Firstly, intra-aortic balloon counterpulsation (IABP) should be performed to improve the child’s cardiac output and coronary artery perfusion pressure, while reducing the left ventricular afterload. If the circulation cannot be corrected or significantly improved, extracorporeal membrane oxygenators (ECMO) are immediately initiated to either replace or partially substitute the child’s cardiopulmonary function, thereby sustaining life and allowing for the potential restoration of the heart, lungs, and other vital organs. At the same time, co-adjuvant continuous renal replacement therapy (CRRT) can provide early life-supporting replacement therapy, and adjuvant medication can ensure adequate rest of the heart, thus maximizing the restoration of cardiac function and improving the prognosis of the child.^7^ In addition to these traditional diagnostic methods, searching for and evaluating the diagnostic value of new biomarkers for PFM is also of vital importance.

microRNAs (miRNAs) pitch in the regulation of genes in cardiac physiology and pathology at the post-transcriptional level.^8^ In the cardiovascular system, miRNAs have been documented to control the functions of various cells, such as endothelial cells, cardiomyocytes, fibroblasts and smooth muscle cells.^9^ Among these, the most extensively studied miRNA is miR-21, which has been proposed as a diagnostic and predictive biomarker for numerous cardiac disorders, like diabetic cardiomyopathy, coronary artery disease, and myocardial ischemic reperfusion injury.^10^ Circulating hsa-miR-21 is a miRNA closely linked with cardiovascular diseases, such as atherosclerosis and coronary heart disease.^11^ Reportedly, mmu-miR-21 serves as a novel therapeutic target for the management of myocarditis.^12^ He et al.^12^ have observed that the mmu-miR-21 level in mice with myocarditis is remarkably abated in comparison to the healthy control mice. Currently, there is a limited number of studies exploring the role of serum circulating hsa-miR-21 in FM pediatric patients, as well as a scarcity of research investigating the clinical significance of serum circulating hsa-miR-21 expression in this population. Based on this context, the objective of this study was to explore serum circulating hsa-miR-21 expression in the serum and to evaluate the clinical value of hsa-miR-21 in FM pediatric patients.

## Materials and methods

### Study subjects

A cohort of 100 FM pediatric patients admitted to The First Affiliated Hospital of Anhui Medical University from March 2020 to March 2024 were retrospectively and continuously selected, of which 7 cases were excluded due to incomplete data. Based on the inclusion and exclusion criteria, an additional 5 cases were excluded, resulting in a final sample size of 88 patients, who were included in the FM group. Additionally, 90 healthy children, who were matched for sex and age with FM pediatric patients and had normal physical examination results during the same period, were selected as the normal control (NC) group. All study subjects had complete clinical data.

## Results

### Comparisons of general clinical baseline data

As reflected by baseline physical characteristics, FM pediatric patients showed decreased SBP and DBP (all *p* < 0.001), elevated HR (*p* < 0.001), and boosted levels of biochemical parameters such as WBC, MYO, B-type natriuretic peptide (BNP), cardiac troponin I (cTnI), creatine kinase isoenzyme (CK-MB), C-reactive protein (CRP), lactate dehydrogenase (LDH), and lactate (Lac) (all *p* < 0.001, Supplementary Table 2).

The FM pediatric patients were allocated into the good prognosis group (*n* = 57) and the poor prognosis group (*n* = 31) based on their outcomes. A comparative analysis was conducted on clinical data between the two groups. No significant difference was observed between the two groups concerning sex, age, BMI, the main clinical symptoms (chest tightness, chest pain, dyspnea, and fever), WBC, cTnI, valvular regurgitation, ventricular wall dyskinesia, pericardial effusion, ventricular enlargement, tachycardia, conduction block, premature beat, abnormal Q wave, or long QT interval (all *p* > 0.05). However, SBP and DBP in the poor prognosis group were lower than in the good prognosis group (all *p* < 0.05). MYO, BNP, CK-MB, CRP, LDH, and Lac levels were raised (all *p* < 0.05), and the LVEF was decreased in the poor prognosis group compared to the good prognosis group (*p* < 0.05) (Supplementary Table 3).

### The serum hsa-miR-21 level was elevated in FM pediatric patients, and was correlated with poor prognoses

**Figure 1:**
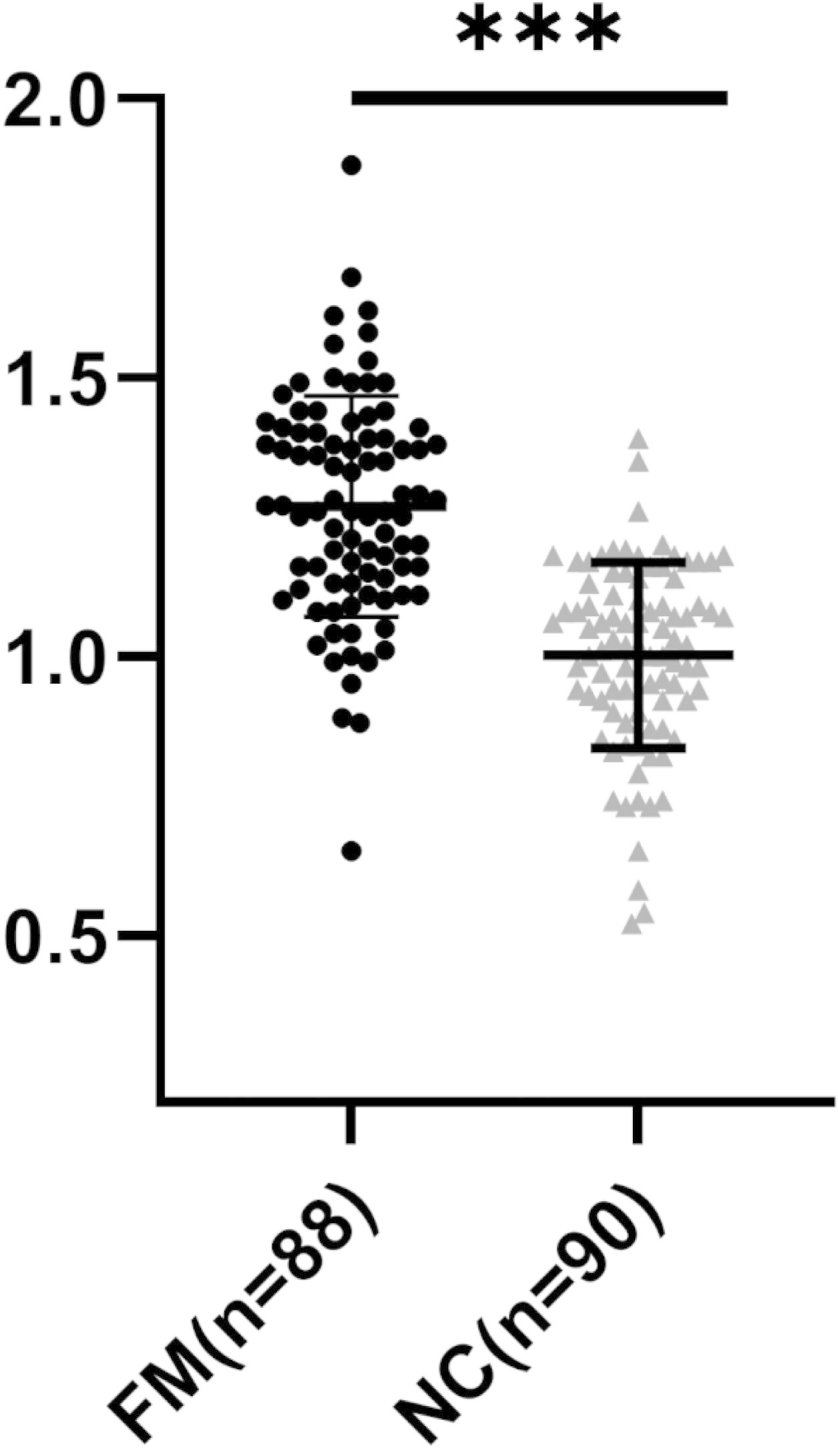
Expression levels of hsa-miR-21 in different groups. hsa-miR-21 expression levels in the serum of pediatric patients in the FM group and children in the NC group were determined to use RT-qPCR. Data were expressed as *x* ± *s*, and inter-group comparisons were conducted using the independent sample *t*-test. ^⁎⁎⁎^*p* < 0.001. (a) Comparison of hsa-miR-21 levels between the FM and NC groups; (b) Comparison of hsa-miR-21 levels between the good and poor prognosis groups.

RT-qPCR was used to determine hsa-miR-21 expression levels in serum of the FM and NC groups, with the results demonstrating that serum hsa-miR-21 expression levels were 1.17 ± 0.30 in pediatric patients in the FM group and 0.21 ± 0.09 in children in the NC group, which were substantially higher in the FM group than in the NC group (*p* < 0.001, Figure 1). Serum hsa-miR-21 expression in the good prognosis group was 1.06 ± 0.28 and was 1.37 ± 0.23 in the poor prognosis group, which was higher in the poor prognosis group than in the good prognosis group (*p* < 0.001, Figure 1). During the treatment period, 1 pediatric patient died, with a mortality rate of 1.14 % and a serum hsa-miR-21 level of 1.89. Two pediatric patients passed away within three months after the end of the treatment, with a mortality rate of 2.30 % and serum hsa-miR-21 levels of 1.91 and 1.67, respectively. Notably, all pediatric patients who died exhibited elevated levels of serum hsa-miR-21.

### Correlation analysis of hsa-miR-21 levels with myocardial injury marker (MYO, BNP, cTnI and CK-MB) levels in FM pediatric patients

**Figure 2:**
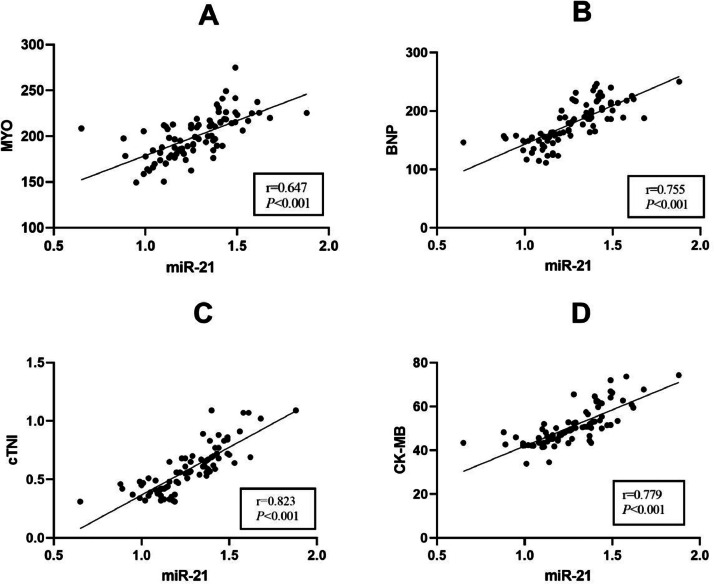
Correlations of hsa-miR-21 with MYO, BNP, cTnI, and CK-MB levels. The correlations of serum hsa-miR-21 levels with MYO (a), BNP (b), cTnI (c), and CK-MB (d) levels in FM pediatric patients were analyzed by Pearson’s correlation coefficient.

As demonstrated by Pearson’s correlation coefficient results, serum hsa-miR-21 levels in FM pediatric patients were significantly positively interrelated with the levels of myocardial injury markers MYO (Figure 2a, *r* = 0.578, *p* < 0.001), BNP (Figure 2b, *r* = 0.516, *p* < 0.001), cTnI (Figure 2c, *r* = 0.639, *p* < 0.001), and CK-MB (Figure 2d, *r* = 0.674, *p* < 0.001) (Figure 2).

### The multivariate regression analysis on independent risk factors for the poor prognosis in FM pediatric patients

To more accurately assess the effect of the hsa-miR-21 level on prognoses of FM pediatric patients, logistic univariate regression analyses were performed, with the good/poor prognosis (0 = No, 1 = Yes) as the dependent variable, and the indicators with *p* < 0.05 in Supplementary Table 3 as the independent variables. It was found that SBP, DBP, hsa-miR-21, MYO, BNP, CK-MB, LVEF, CRP, Lac, and LDH were the influencing factors for poor prognoses in FM pediatric patients (Table 1). The influencing factors in the univariate analysis, including SBP, DBP, hsa-miR-21, MYO, BNP, CK-MB, LVEF, CRP, Lac, and LDH, were included as independent variables in the logistic multivariate regression analysis. The results showed that serum hsa-miR-21 (*p* = 0.030, OR = 68.990, 95 % CI = 1.498–3177.094), CK-MB (*p* = 0.013, OR = 1.377, 95 % CI = 1.071–1.772), LVEF (*P* = 0.024, OR = 0.581, 95 % CI = 0.362–0.932), CRP (*p* = 0.041, OR = 1.070, 95 % CI = 1.003–1.141), Lac (*p* = 0.021, OR = 11.570, 95 % CI = 1.444–92.671), and LDH (*p* = 0.013, OR = 1.032, 95 % CI = 1.007 −1.058) were independent risk factors for poor prognoses in FM pediatric patients after excluding potential confounders. For every 1-unit elevation in serum circulating hsa-miR-21, the risk of poor prognoses in FM pediatric patients increased 68.990-fold.

**Table 1:** Univariate and multivariate regression analyses on independent risk factors for poor prognosis in FM pediatric patients.

	Logistic univariate regression analysis			Logistic multivariate regression analysis		
	*P* value	OR value	95 % CI	*P* value	OR value	95 % CI
SBP	0.043	0.953	0.909–0.999	0.055	0.885	0.781–1.003
DBP	0.046	0.938	0.881–0.999	0.513	0.953	0.826–1.100
hsa-miR-21	<0.001	115.009	10.790–1225.874	0.03	68.99	1.498–3177.094
MYO	0.029	1.022	1.002–1.041	0.306	1.021	0.981–1.063
BNP	0.039	1.017	1.001–1.034	0.113	1.026	0.994–1.058
CK-MB	<0.001	1.243	1.109–1.394	0.013	1.377	1.071–1.772
LVEF	0.026	0.773	0.617–0.969	0.024	0.581	0.362–0.932
CRP	0.002	1.047	1.017–1.079	0.041	1.07	1.003–1.141
Lac	0.019	3.004	1.197–7.539	0.021	11.57	1.444–92.671
LDH	0.016	1.016	1.003–1.030	0.013	1.032	1.007–1.058

MYO, myoglobin; BNP, B-type natriuretic peptides; CK-MB, Creatine Kinase-MB; LVEF, left ventricular ejection fractions; SBP, systolic blood pressure; DBP, diastolic blood pressure; CRP, C-reactive protein; LDH, lactate dehydrogenase; Lac, Lactate.

### Diagnostic value of hsa-miR-21 levels for poor prognosis in FM pediatric patients

**Figure 3:**
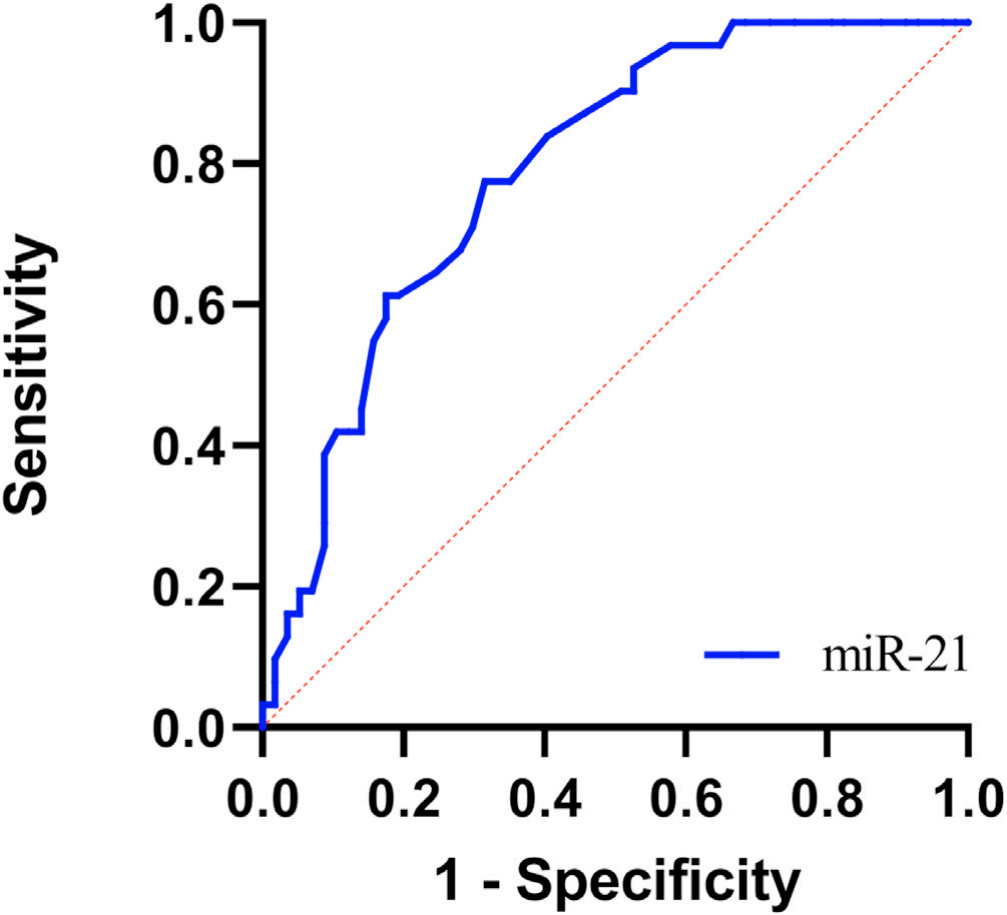
ROC curve of hsa-miR-21 predicting poor prognosis in FM pediatric patients.

Given the differential expression of hsa-miR-21 in the good and poor prognosis groups, the authors speculated that hsa-miR-21 might serve as a potential biomarker for predicting poor prognosis in FM pediatric patients. Therefore, the authors further plotted ROC curves to evaluate the diagnostic value of hsa-miR-21 in FM pediatric patients. The AUC of serum hsa-miR-21 levels for predicting the poor prognosis in FM pediatric patients was 0.790, with a sensitivity of 58.1 % and a specificity of 87.7 % (Figure 3). The positive predictive value (PPV) and negative predictive value (NPV) of serum hsa-miR-21 for the poor prognosis of pediatric FM patients were 74.07 % and 81.97 %, respectively, both of which were lower than 90 % (Supplementary Table 4), demonstrating that hsa-miR-21 aided in predicting the poor prognosis of FM pediatric patients to some extent. The diagnostic accuracy was, however, limited and hsa-miR-21 should be combined with other clinical indicators or further examination results for comprehensive judgment.

## Discussion

Currently, FM lacks validated biomarkers and well-established therapeutic interventions.^13^ As the potential diagnostic biomarkers, the effectiveness of miRNAs in FM remains largely unexplored.^14^ Consequently, the present study explored the expression and clinical value of hsa-miR-21 in FM pediatric patients.

Biomarkers can aid in both the diagnosis of FM and the assessment of disease severity. cTnI is one of the most valuable and sought-after biomarkers for the detection of various forms of myocardial injury in contemporary clinical practice.^15^ Moreover, remarkably enhanced plasma CK-MB and WBC serve as indirect indicators of myocardial dysfunction.^1^ A meta-analysis conducted by Wang et al. has revealed that patients with FM exhibit markedly reduced SBP and DBP, an elevated CK level, and a reduced LVEF compared to non-FM patients.^16^ MYO is a cytoplasmic hemoglobin found in skeletal muscle fibers and cardiomyocytes, with its level increasing in response to myocardial and skeletal muscle injury.^17^ Elevated levels of BNP are observed in FM patients compared to those with pericarditis or acute myocarditis.^18^ CRP is the most frequently used acute-phase response indicator in clinical settings, and changes in its expression can rapidly reflect minor alterations in the circulatory system. Additionally, CRP is less affected by anti-inflammatory and hormonal medications, allowing it to more sensitively reflect the severity of myocardial injury.^19^ Blood Lac is a product of anaerobic glycolysis closely tied up with movement and metabolism, which cannot only detect the presence or the absence of disorders in the respiratory and circulatory systems in the human body but also reflect disease severity.^19^ Zhao et al.^20^ have shown that Lac and LDH are risk factors for FM pediatric patients. Similarly, the present study also revealed that FM pediatric patients exhibited reduced SBP and DBP, up-regulated HR, and elevated levels of biochemical markers WBC, MYO, BNP, cTnI, CK-MB, CRP, LDH, and Lac. Subsequently, the authors compared FM-related parameters by assigning FM patients into the poor and good prognosis groups based on the prognosis. A prior study demonstrated that a decrease in SBP at an early stage is independently linked to a poor prognosis in patients with acute heart failure.^21^ During a six-month follow-up period, an elevated level of MYO (greater than 705.8 ng/mL) has been identified as one of the independent predictors of poor prognosis in male patients with acute myocardial infarction.^22^ A twofold increase in CK-MB following transfemoral transcatheter aortic valve replacement serves as a surrogate marker for unfavorable long-term outcomes in myocardial injury patients.^23^ Intriguingly, the present study data unveiled that compared with FM pediatric patients with favorable prognosis, those with poor prognosis exhibited abated SBP and DBP, raised WBC, BNP, CK-MB, CRP, LDH, and Lac levels, alongside a reduction in LVEF.

In patients and animal models with acute myocardial infarction, miR-21 is highly expressed in circulation as a result of ischemia and injury.^24^ Furthermore, miR-21 has been demonstrated to be up-regulated in inflammatory heart diseases.^25^ Innovatively, the present study data indicated that serum hsa-miR-21 expression was elevated in FM pediatric patients, as well as in FM pediatric patients with poor prognoses. Notably, plasma miR-21 expression is positively interrelated with BNP in patients with pregnancy-induced hypertension complicated with heart failure.^26^ cTnI level has been suggested to be positively correlated with miR-21 in patients with atherosclerosis.^27^ Moreover, plasma levels of CK-MB and cTnI also demonstrate positive correlations with plasma miR-21 levels in patients with acute myocardial infarction.^28^ Innovatively, the authors found that hsa-miR-21 levels were favorably correlated with MYO, BNP, cTnI, and CK-MB levels. Furthermore, hsa-miR-21, CK-MB, LVEF, CRP, Lac, and LDH were found to be independent risk factors for poor prognosis in FM pediatric patients. Additionally, for every 1-unit elevation of serum circulating hsa-miR-21, children with FM had a 68.990-fold increased risk of poor prognoses. The prognostic potential of miR-21 has been shown in various diseases like hypertension, liver cirrhosis, and coronary artery disease,^29^^,^^30^ but the prognostic value of hsa-miR-21 for PFM remains largely unknown. To fill this gap, the authors plotted the ROC curve to assess the prognostic value of hsa-miR-21 in FM pediatric patients, with the results indicating that hsa-miR-21 level had diagnostic value for the prognosis of FM pediatric patients. PPV and NPV of serum hsa-miR-21 for poor prognoses of pediatric FM patients were 74.07 % and 81.97 %, respectively, both of which were lower than 90 %, demonstrating that hsa-miR-21 aided in predicting the poor prognosis of FM pediatric patients to some extent. However, the diagnostic accuracy is limited, and hsa-miR-21 should be combined with other clinical indicators or further examination results for comprehensive judgment. During the treatment period, 1 pediatric patient died, with a mortality rate of 1.14 % and a serum hsa-miR-21 level of 1.89. Two pediatric patients passed away within three months after the end of the treatment, with a mortality rate of 2.30 % and serum hsa-miR-21 expression of 1.91 and 1.67, respectively. Notably, all pediatric patients who died exhibited elevated levels of serum hsa-miR-21. The above results indicated that the measurement of serum hsa-miR-21 levels might play a pivotal role in the clinical care of FM pediatric patients and in the formulation and selection of personalized treatment strategies.

All in all, the present study found that serum hsa-miR-21 was up-regulated in FM pediatric patients, with its level positively correlating with MYO, BNP, cTnI and CK-MB, and that hsa-miR-21 expression could predict the prognosis of FM pediatric patients to some extent. However, the diagnostic value is limited and hsa-miR-21 should be combined with other clinical indicators or further examination results for comprehensive judgment.

Nonetheless, there are still some limitations that need to be considered in this study. Firstly, the sample size was relatively small, and pertinent influencing factors, such as dietary habits and family medical history of the participants, were not controlled for. Secondly, this study only focused on the correlations between the expression of hsa-miR-21 and the levels of MYO, BNP, cTnI and CK-MB, leaving it unclear whether additional factors are associated with hsa-miR-21 expression, or whether hsa-miR-21 influences the levels of MYO, BNP, cTnI and CK-MB through the modulation of other variables. Furthermore, the mechanisms of the action of hsa-miR-21 expression on the occurrence and development of FM are not well understood. In the future, the authors will increase the sample size and conduct multicenter studies to elucidate the role of hsa-miR-21 expression in the diagnosis and assessment of FM. Additionally, further investigation into the regulatory mechanisms of hsa-miR-21 and its relationship with target genes is warranted. Finally, it is essential to comprehensively consider influencing factors in future studies.

## Ethics approval and consent to participate

The study was reviewed and ratified by the Ethics Committee of The First Affiliated Hospital of Anhui Medical University and was in accordance with the Declaration of Helsinki. Informed consent was obtained from the legal guardians of all pediatric patients involved in the study.

## Consent for publication

Not applicable.

## Availability of data and materials

All data included in this study are available upon request by contact with the corresponding author.

## Funding

No funding was received for this study.

## Authors’ contributions

Guarantor of integrity of the entire study: Wenwen Wang; Study concepts: Wenwen Wang; Study design: Wenwen Wang; Definition of intellectual content: Jingmin Sun and Jing Li; Literature research: Wenwen Wang; Clinical studies: Jingmin Sun and Jing Li; experimental studies: Wenwen Wang; data acquisition: Wenwen Wang and Jing Li; data analysis: Jingmin Sun and Wenwen Wang; statistical analysis: Wenwen Wang; manuscript preparation: Wenwen Wang; manuscript editing: Jing Li; manuscript review: Jingmin Sun.

## Conflicts of interest

The authors declare no conflicts of interest.

## Appendix Supplementary materials

Image, application 1

## Footnotes

Supplementary material associated with this article can be found in the online version at doi:10.1016/j.jped.2025.04.007.
