# Supplementary material for: Serum hsa-miR-21 expression and its clinical value in pediatric patients with fulminant myocarditis
Source: J Pediatr (Rio J). 2025 May 28;101(5):101409. doi: 10.1016/j.jped.2025.04.007 (PMC12495583; doi:10.1016/j.jped.2025.04.007)
Supplement: Supplementary file 1 [file mmc1.docx]

**JPED-D-24-00567_Supplementary Materials**

**Supplementary Table 1** Primer sequences.

| Primer | Primer sequence(5’-3’) |
| --- | --- |
| hsa-miR-21 (forward) | ACACTCCAGCTGGGTAGCTTATCAGACTGA |
| hsa-miR-21 (reverse) | CTCAACTGGTGTCGTGGAGTCGGCAATTCAGTTGAGTCAACATC |
| cel-miR-39-3p (forward) | GACTTCATCACCGGGTGTAAATC |
| cel-miR-39-3p(reverse) | TATCGTTGTTCTCCACTCCTTGAC |

**Supplementary Table 2** Comparisons of general data of the two groups.

| Parameters | Normal range | FM group (n = 88) | NC group (n =90) | t/X² value | *P* value |
| --- | --- | --- | --- | --- | --- |
| General data |  |  |  |  |  |
| Male (cases, %) |  | 40 (45.45) | 45 (50.00) | 0.541 | 0.544 |
| Age (years) | < 18 | 9.10 ± 2.83 | 9.67 ± 3.31 | 0.356 | 0.197 |
| Baseline physical characteristics |  |  |  |  |  |
| BMI | 7.00-24.00 | 18.32 ± 3.24 | 18.20 ± 2.76 | 0.277 | 0.782 |
| SBP (mmHg) | 90-135 | 98.38 ± 10.12 | 108.91 ± 5.93 | 8.485 | < 0.001 |
| DBP (mmHg) | 40-80 | 59.07 ± 8.01 | 69.55 ± 8.90 | 8.252 | < 0.001 |
| HR (times/min) | 70-100 | 76.97 ± 13.38 | 89.36 ± 4.65 | 8.288 | < 0.001 |
| Biochemical index |  |  |  |  |  |
| WBC (10^9^/L) | 4-10 | 13.68 ± 2.51 | 5.40 ± 2.54 | 22.28 | < 0.001 |
| MYO (μg/L) | 0-80 | 197.10 ± 24.16 | 53.22 ± 13.43 | 48.24 | < 0.001 |
| BNP (pg/mL) | 0-100 | 182.31 ± 28.68 | 61.47 ± 11.86 | 36.86 | < 0.001 |
| cTnI (ng/mL) | 0-1.95 | 0.58 ± 0.20 | 0.28 ± 0.12 | 12.62 | < 0.001 |
| CK-MB (U/L) | 0-25 | 50.15 ± 6.17 | 13.22 ± 2.96 | 51.10 | < 0.001 |
| CRP(mg/L) | 0-10 | 40.29 ± 17.34 | 1.74 ± 0.45 | 21.10 | < 0.001 |
| LDH(U/L) | 80-280 | 291.90 ± 38.35 | 112.24 ± 17.79 | 40.23 | < 0.001 |
| Lac(mmol/L) | 0.5-1.7 | 2.64 ± 0.50 | 0.26 ± 0.05 | 44.54 | < 0.001 |

BMI, body mass index; WBC, white blood cell; MYO, myoglobin; BNP, B-type natriuretic peptides; cTnI, Cardiac Troponin; CK-MB, Creatine Kinase-MB; SBP, systolic blood pressure, DBP, diastolic blood pressure; HR, heart rate; CRP, C-reactive protein; LDH, lactate dehydrogenase; Lac, Lactate. Count data were depicted as percentages, and normally distributed measurement data were expressed as x ± s and examined using independent sample *t* test. p < 0.05 indicated statistically significant differences.

**Supplementary Table 3** Comparisons of clinical data between the good prognosis and poor prognosis groups.

| Parameters | Good prognosis group (n = 57) | Poor prognosis group (n = 31) | t/X² value | *P* value |
| --- | --- | --- | --- | --- |
| General data |  |  |  |  |
| Male (cases, %) | 27 (47.40) | 13 (41.90) | 0.239 | 0.625 |
| Age (years) | 9.40 ± 2.64 | 8.41 ± 3.10 | 1.571 | 0.120 |
| Basic physical characteristics |  |  |  |  |
| BMI | 18.76 ± 3.50 | 17.50 ± 2.56 | 1.756 | 0.081 |
| SBP (mmHg) | 100.00 ± 11.10 | 95.38 ± 7.24 | 2.092 | 0.039 |
| DBP (mmHg) | 60.35 ± 9.31 | 56.71 ± 4.66 | 2.073 | 0.041 |
| HR (times/min) | 121.79 ± 10.42 | 126.77 ± 14.72 | 1.846 | 0.068 |
| Clinical symptoms |  |  |  |  |
| Chest tightness (cases, %) | 29 (50.88) | 13 (41.94) | 0.644 | 0.422 |
| Chest pain (case, %) | 17 (29.82) | 9 (29.03) | 0.006 | 0.938 |
| Dyspnea (case, %) | 8 (14.04) | 3 (9.68) | 0.349 | 0.555 |
| Fever (case, %) | 30 (52.63) | 16 (51.61) | 0.008 | 0.927 |
| Biochemical indicators |  |  |  |  |
| WBC (10^9^/L) | 13.29 ± 1.83 | 14.38 ± 3.341 | 1.971 | 0.052 |
| MYO (μg/L) | 192.82 ± 22.00 | 204.88 ± 25.73 | 2.290 | 0.024 |
| BNP (pg/mL) | 177.5 ± 21.27 | 191 ± 37.69 | 2.141 | 0.035 |
| cTnI (ng/mL) | 0.54 ± 0.13 | 0.61 ± 0.24 | 3.348 | 0.061 |
| CK-MB (U/L) | 48.07 ± 5.54 | 53.98 ± 5.43 | 4.810 | < 0.001 |
| CRP (mg/L) | 35.89 ± 16.51 | 48.38 ± 16.07 | 3.421 | 0.001 |
| LDH (U/L) | 284.21 ± 30.43 | 305.90 ± 47.14 | 2.615 | < 0.001 |
| Lac (mmol/L) | 2.54 ± 0.50 | 2.82 ± 0.55 | 3.421 | 0.001 |
| Ultrasoundcardiogram indicators |  |  |  |  |
| LVEF (%) | 33.63 ± 2.21 | 32.52 ± 1.90 | 2.344 | 0.021 |
| Valvular regurgitation (cases, %) | 37 (64.91) | 23 (74.19) | 0.797 | 0.372 |
| Ventricular wall dyskinesia (case, %) | 26 (45.61) | 17 (54.83) | 0.684 | 0.408 |
| Pericardial effusion (case, %) | 16 (28.07) | 9 (29.03) | 0.009 | 0.924 |
| Ventricular enlargement (case, %) | 25 (43.86) | 16 (51.61) | 0.485 | 0.486 |
| ECG indicators |  |  |  |  |
| Tachycardia (cases, %) | 39 (68.42) | 24 (77.41) | 0.799 | 0.371 |
| Conduction block (case, %) | 15 (26.32) | 10 (32.25) | 0.349 | 0.555 |
| Premature beat (case, %) | 5 (8.77) | 2 (6.45) | 0.148 | 0.701 |
| Abnormal Q wave (%) | 12 (21.05) | 5 (16.13) | 0.312 | 0.576 |
| Long QT interval (case, %) | 6 (10.53) | 3 (9.68) | 0.016 | 0.900 |

BMI, body mass index; WBC, white blood cell; MYO, myoglobin; BNP, B-type natriuretic peptides; cTnI, Cardiac Troponin; CK-MB, Creatine Kinase-MB; LVEF, left ventricular ejection fractions; SBP, systolic blood pressure; DBP, diastolic blood pressure; HR, heart rate; ECG, electrocardiogram; CRP, C-reactive protein; LDH, lactate dehydrogenase; Lac, Lactate. The count data were expressed as percentages. Normally distributed measurement data were presented as x ± s and examined using the independent sample *t* test. Comparisons between groups were conducted using the Mann-Whitney U test. *P* < 0.05 indicated a statistically significant difference.

**Supplementary Table 4** Analyses of PPV and NPV of serum hsa-miR-21 for the poor prognosis in FM pediatric patients.

| hsa-miR-21 > 1.29 (n = 27) | | | hsa-miR-21 ≤ 1.29 (n = 61) | | |
| --- | --- | --- | --- | --- | --- |
| True positive (cases) | False positive (cases) | PPV (%) | True negative (cases) | False negative (cases) | NPV (%) |
| 20 | 7 | 74.07 | 50 | 11 | 81.97 |

PPV, Positive Predictive Value; NPV, Negative Predictive Value.

**Supplementary materials**

**Ethics statement**

The study was reviewed and ratified by the Ethics Committee of The First Affiliated Hospital of Anhui Medical University and was in accordance with the Declaration of Helsinki. Informed consent was obtained from the legal guardians of all pediatric patients involved in the study.

**Inclusion and exclusion criteria**

The inclusion criteria were as follows: (1) in accordance with the diagnostic criteria in the American Heart Association 2020 Scientific Statement on FM : i) the presence of signs or symptoms of viral upper respiratory tract infection or enteroviral infection recently, and presence of cardiovascular symptoms; ii) a rapid onset of heart failure in the absence of underlying cardiovascular diseases; iii) presence of shock, electrical instability, or the rapid progression of conduction abnormalities; (2) presence of severe heart failure immediately following an antecedent infection, classification into New York Heart Association class IV, and presence of malignant arrhythmia, or cardiogenic shock that required positive inotropic/vasoactive medications and triple-integrated therapy (IABP, ECMO, CRRT) (7); (3) age < 18 years; (4) complete clinical data.

Exclusion criteria included: (1) presence of congenital atrioventricular block, congenital bundle branch block, ion channel disease, orthostatic intolerance, beta receptor hyperfunction, and drug-induced electrocardiogram changes; (2) presence of congenital heart disease, non-ischemic cardiomyopathy, myocardial infarction, and cardiac tumors; (3) presence of congenital or acquired autoimmune diseases; (4) a recent history of surgical trauma or blood transfusion, (5) complication of metabolic diseases (such as hyperthyroidism and glycogen storage disease and other genetic metabolic diseases).

**Data and sample collection**

The following clinical baseline data were acquired from all enrolled subjects: (1) general information: sex and age of all children; (2) baseline physical characteristics: body mass index (BMI), systolic blood pressure (SBP), diastolic blood pressure (DBP), and heart rate (HR) on the day of admission or physical examination of all children; (3) biochemical parameters: white blood cell (WBC), myoglobin (MYO), BNP, cTnI and CK-MB levels; (4) clinical symptoms: mainly a series of discomfort complaints of the pediatric patients at the time of admission, including chest tightness, chest pain, dyspnea, fever, etc.; (5) ultrasoundcardiogram indexes: cardiac ultrasound data of the pediatric patients on admission, including left ventricular ejection fraction (LVEF), valvular regurgitation, ventricular wall dyskinesia, pericardial effusion, ventricular enlargement, etc.; (6) ECG indicators: arrhythmia type and other abnormalities in the pediatric patients at the time on admission, including tachycardia, conduction block, premature beat, abnormal Q wave, and long QT interval.

For sample collection, 6 mL of fasting venous serum samples were obtained from healthy children on the day of physical examination and FM pediatric patients on the morning of admission for reverse transcription-quantitative polymerase chain reaction (RT-qPCR) detection.

**RT-qPCR**

Serum samples preserved in 1.5-mL RNAase-free microcentrifuge tubes were utilized for RNA concentration determination within 1 week. Total serum RNA was extracted using TRIzol reagent (Thermo Fisher Scientific, Waltham, MA, USA). hsa-miR-21 was reversely transcribed using the Rneasy reagent (Qiagen, Hilden, Germany), and the procedure was strictly implemented as per the kit instruction. The amount of synthesized complementary DNA (cDNA) was determined using a Nano-Drop 2000 spectrophotometer (Thermo Fisher Scientific). The qPCR reaction was performed on a fluorescence qPCR instrument (Bio-Rad, Hercules, CA, USA). Reaction system: 10 μL SYBR Mix,8 μL H_2_O, 0.5 μL upstream and downstream primers, 1 μL cDNA template. The reaction conditions were as below: initial denaturation at 95°C for 5 min, followed by 40 cycles of 95°C for 15 s, 60°C for 30 s, 72°C for 1 min, and 72°C for 10 min. The exogenous external reference was cel-miR-39-3p. The primer sequences are listed in Supplementary Table 1 (18). The relative expression of serum hsa-miR-21 was quantified by the 2^-ΔΔCt^ method.

**Treatment protocol**

After admission, FM pediatric patients were given general treatment (monitoring, bed rest, oxygen inhalation, sedation, control of infusion volume and speed), nutritional myocardial therapy, and supportive therapy such as immunoglobulin and glucocorticoid. General pediatric patients received antiviral therapy and high-dose gammaglobulin shock therapy (1 g/kg per day for 2 days), supplemented with high-dose vitamin C and sodium creatine phosphate to nourish the myocardium. Among them, 41 FM pediatric patients who developed heart failure and cardiogenic shock were administered dopamine and dobutamine following volume expansion to improve circulation, and a maintenance dose of digoxin, captopril, and diuretics was introduced at the later stage to improve the cardiac function and reduce the cardiac load. The 20 pediatric patients with high atrioventricular block were simultaneously given methylprednisolone (10 mg/kg per day for 3 days), which was subsequently changed to prednisone (orally), and was tapered off according to the condition in about 1 month. Nine pediatric patients developed severe cardiovascular dysfunction requiring interventions such as mechanical ventilation, temporary or permanent pacemaker implantation, ECMO, and CRRT.

**Prognosis**

ECG, troponin recovery, changes in clinical symptoms, and death of FM pediatric patients from the beginning of treatment to within three months after the end of the treatment were compared. Then, they were divided into the good prognosis group, which included patients whose clinical symptoms had either disappeared or improved and whose ECG or troponin levels had returned to normal, and the poor prognosis group, which comprised pediatric patients who died, had persistent ventricular tachycardia or ventricular fibrillation, underwent re-admission to hospital for heart failure, and had recurrent cardiomyopathy. The good prognosis group consisted of 57 cases, while the poor prognosis group included 31 cases.

**Statistical analysis**

Data were statistically analyzed and graphed using SPSS 21.0 (IBM, Armonk, NY, USA) and GraphPad Prism 6.0 (GraphPad Software, San Diego, CA, USA) software. The normal distribution of data was examined using the Shapiro-Wilk test. Non-normally distributed measurement data were expressed in quartiles, and inter-group comparisons were conducted using the Mann-Whitney U test. Quantitative data that conformed to normal distribution were presented as mean ± standard deviation (x ± s), and an independent sample *t* test was adopted for inter-group comparisons. Count data were depicted as cases and percentages, and the Chi-square test was employed for inter-group comparisons. Logistic regression analysis was implemented to analyze hsa-miR-21 levels and clinical indicators. The correlations of the hsa-miR-21 level with some clinical indicators were analyzed by Pearson’s correlation coefficient. The receiver operating characteristic (ROC) curve was utilized to evaluate the diagnostic value of parameters, the cut-off values were obtained, and the area under the ROC curve (AUC) of hsa-miR-21 was analyzed. Differences were considered to be significant at p < 0.05.
